# Assessment and Management of Substance Misuse Among Patients With Psychosis at a Mental Health Inpatient Unit in CNTW NHS Foundation Trust: An Audit

**DOI:** 10.1192/bjo.2025.10657

**Published:** 2025-06-20

**Authors:** Mithun Pulichumakal Devasia, Gary Martin, Pooja Thakrar, Rana Shahzaib, Adebayo Erinfolami

**Affiliations:** 1 Cumbria Northumberland Tyne and Wear NHS Foundation Trust, Newcastle, United Kingdom; 2 Belfast Heath and Social Care Trust, Belfast, United Kingdom

## Abstract

**Aims:** The audit aims to check compliance with regards to assessment and management of substance misuse among patients admitted with psychosis in male wards, St George’s Park hospital. The following National Institute of Clinical Excellence (NICE) guideline was considered for the audit: Coexisting Severe Mental Illness (Psychosis) and Substance Misuse: Assessment and Management in Healthcare Settings – CG 120 (published on March 23, 2011).

**Methods:** All patients with diagnosis of psychosis and any comorbid substance use history admitted as inpatients in the wards from a period of July 1 to July 31 2024 were considered for the audit. Only patients who were on the wards for a minimum duration of 7 days were included.

A total of 37 patients were found as suitable. Patient progress notes and discharge letters were reviewed to check compliance with standards.

**Results:** Our results showed areas of both good compliance and poor compliance with regards to the standard. We found that 88.8% of patients were assessed for comorbid substance use during the first week of admission by the treatment team. In 62.06% of patients, there was evidence of involvement for specialist drug and alcohol services on admission and in the management of withdrawal symptoms. For 87.5% of patients, it was evident that policies were followed with regards to search procedures, visiting arrangements, planning and reviewing leaves, regular drug and alcohol testing and other security measures. However, only 42.8% compliance was noted regarding involvement with specialist substance use services on discharge.

**Conclusion:** The audit has demonstrated that wards showed good compliance to NICE guidelines around assessment of substance use and in following policies regarding measures to check for substance use in ward settings. However lower compliance was evident with regards to involvement of specialist substance misuse services during both admission and discharge. Following the outcome of audit and presentation in local team meetings, it was decided to initiate a service evaluation to look at barriers regarding involvement of specialist drug and alcohol services along with an educational programme for professionals especially nursing staff regarding the need of screening and referral for people with comorbid substance use in psychosis. A re-audit is planned after a period of 6 months.

